# Supplementary material for: Use of nanosphere self-assembly to pattern nanoporous membranes for the study of extracellular vesicles
Source: Nanoscale Adv. 2020 May 12;2(10):4427–36. doi: 10.1039/d0na00142b (PMC7943038; doi:10.1039/d0na00142b)
Supplement: NA-002-D0NA00142B-s002 [file NA-002-D0NA00142B-s002.pdf]

Electronic Supplementary Material (ESI) for Nanoscale Advances.
This journal is © The Royal Society of Chemistry 2020

Use of Nanosphere Self-Assembly to Pattern Ultrathin Membranes for the Study of Extracellular Vesicles

Authors: Marcela Mireles,[1,2] Cody Soule,[1] Mehdi Dehghani,[1] and Thomas R. Gaborski[1,2]

1. Department of Biomedical Engineering, Rochester Institute of Technology, Rochester, NY, USA

2. Department of Biomedical Engineering, University of Rochester, Rochester, NY, USA

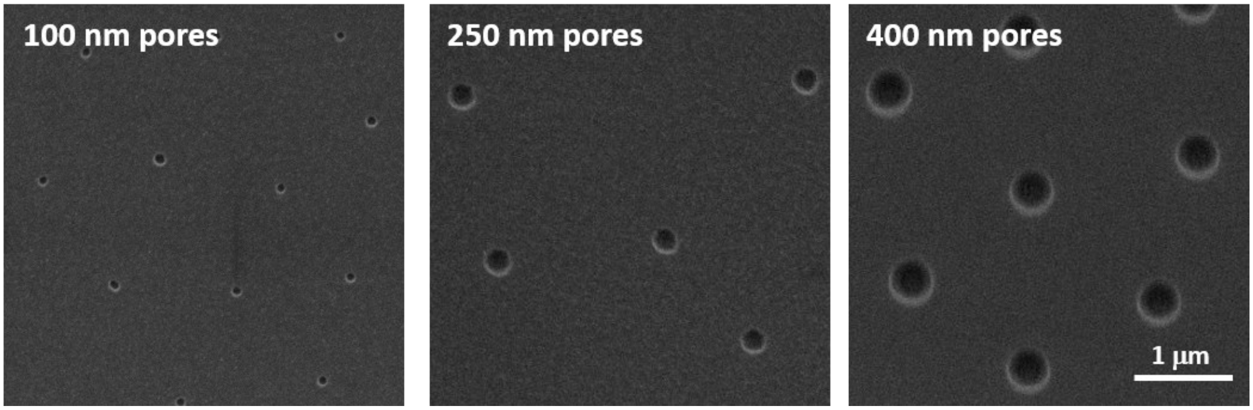

Figure S1. SiO_2 membranes. Top-vie electron micrographs taken from three different SiO_2 membranes fabricated following the same procedure described for SiN in the main manuscript.

In this case, the SiO_2 was deposited by plasma enhanced chemical vapor deposition in an Applied Materials P500 tool at 390 ^{o}C and a working pressure of 1.2 kPa using tetraethoxysilane (TEOS) as precursor. After SiO_2 deposition, the stack (Si/ZnO/SiO_2) was annealed at 600 ^{o}C under N_2 flow for 1 h for stress stabilization. This SiO_2 deposition and subsequent annealing has been previously found to yield a tensile film stress of 150 MPa, stable over time.[1]

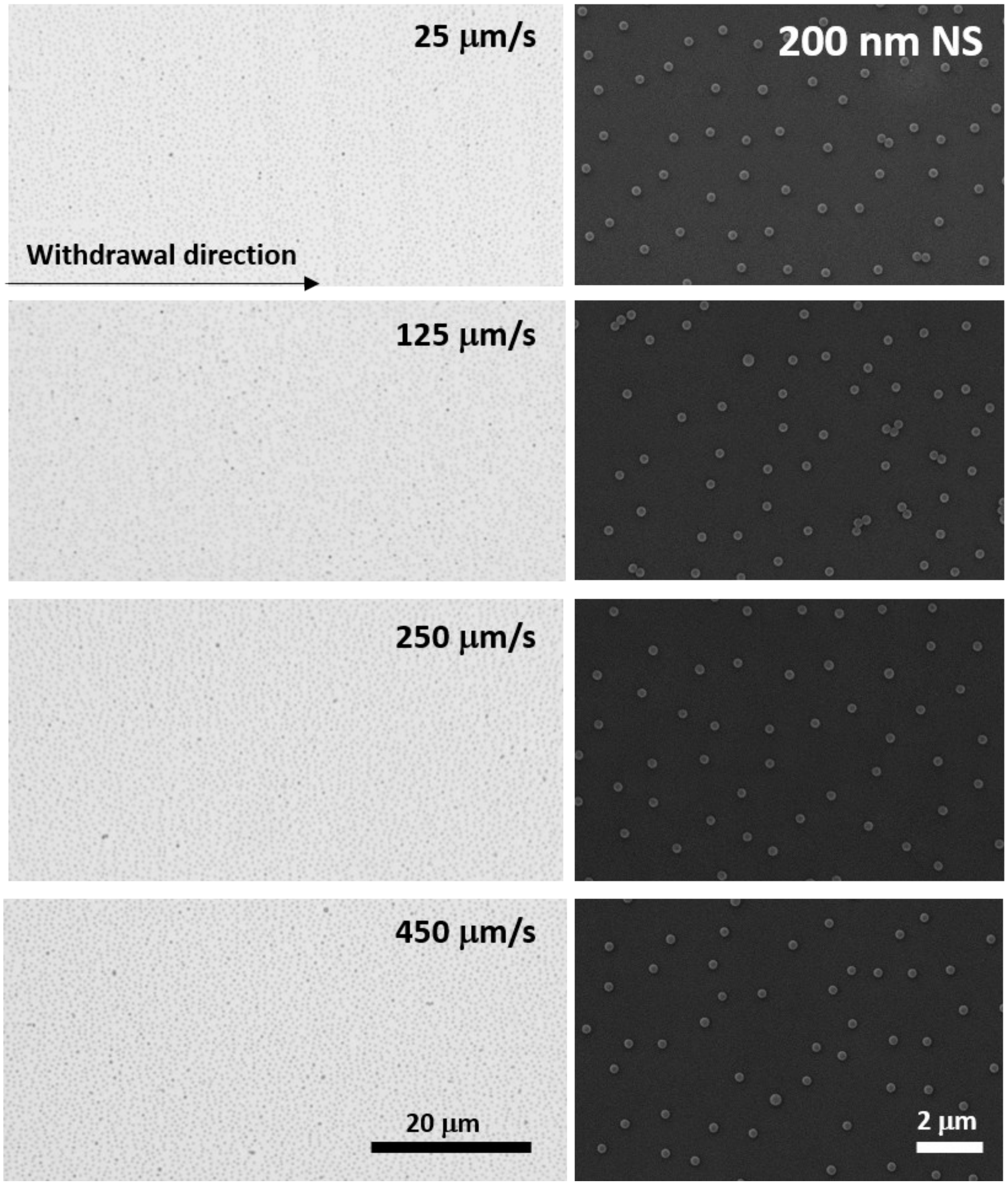

Figure S2. Withdrawal speed optimization. Optical and electron micrographs taken from 200 nm self-assembled monolayers transferred onto a substrate at different withdrawal speeds.

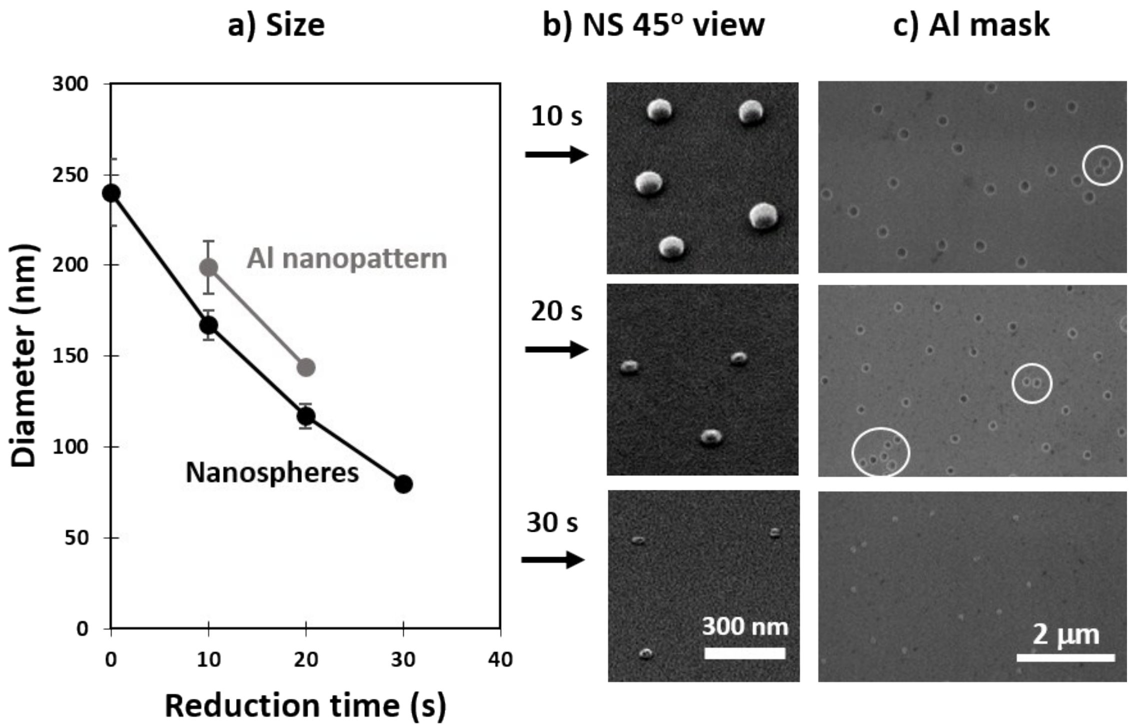

Figure S3. Nanosphere size reduction. a) diameter of the reduced nanospheres (200 nm nominal size) and the resulting nanopores in the Al mask, b) tilted-view of the reduced nanospheres, and c) the resulting pores in the Al mask and unmerging of pores.

The Al mask produced after 10 s of nanosphere reduction presents a doublet (circled in white). When reducing the nanospheres for 20 s two individual pores are observed in close proximity, these constitute a doublet that has been unmerged after reducing the size of the nanospheres.

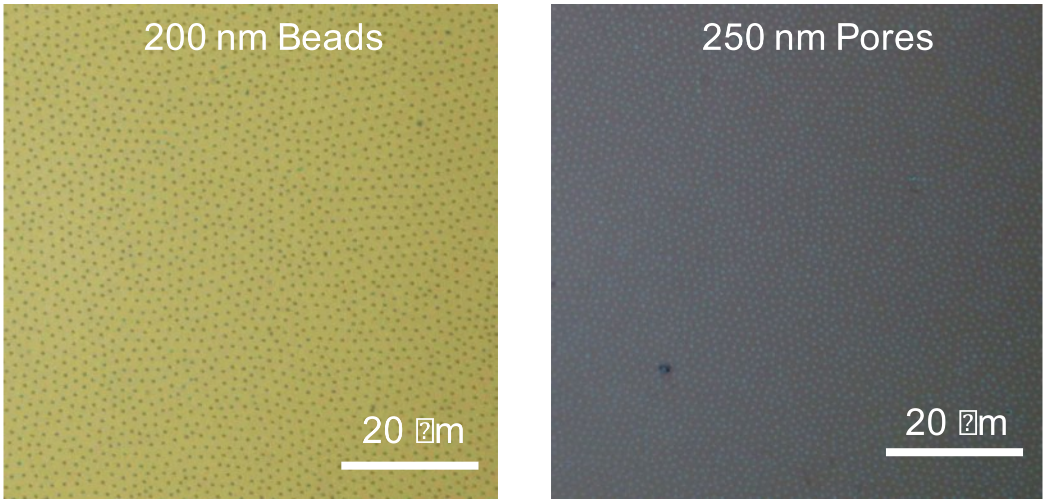

Figure S4. Optical images of beads and corresponding NSL membrane. 100x magnification optical microscopy images of 200 nm beads on a substrate and the resulting 250 nm pores following etching mask fabrication and nanoporous pattern transfer into the SiN membrane.

Supporting video 1. Membrane lift-off: following immersion in 1M HCl for sacrificial film removal, an ultrathin nanoporous silico nitride membrane is released from its supporting substrate, Si wafer, with the use of tweezers.

(1) Carter, R. N.; Casillo, S. M.; Mazzocchi, A. R.; DesOrmeaux, J.-P. S.; Roussie, J. A.; Gaborski, T. R. Ultrathin Transparent Membranes for Cellular Barrier and Co-Culture Models. *Biofabrication* **2017**, *9*, 15019.
